# Research on Deep Learning Detection Model for Pedestrian Objects in Complex Scenes Based on Improved YOLOv7

**DOI:** 10.3390/s24216922

**Published:** 2024-10-29

**Authors:** Jun Hu, Yongqi Zhou, Hao Wang, Peng Qiao, Wenwei Wan

**Affiliations:** School of Electrical and Mechanical Engineering and Vehicle Engineering, East China Jiaotong University, Nanchang 330013, China

**Keywords:** pedestrian detection, YOLOv7, Convolutional Block Attention Module, Deformable ConvNets v2, dynamic head

## Abstract

Objective: Pedestrian detection is very important for the environment perception and safety action of intelligent robots and autonomous driving, and is the key to ensuring the safe action of intelligent robots and auto assisted driving. Methods: In response to the characteristics of pedestrian objects occupying a small image area, diverse poses, complex scenes and severe occlusion, this paper proposes an improved pedestrian object detection method based on the YOLOv7 model, which adopts the Convolutional Block Attention Module (CBAM) attention mechanism and Deformable ConvNets v2 (DCNv2) in the two Efficient Layer Aggregation Network (ELAN) modules of the backbone feature extraction network. In addition, the detection head is replaced with a Dynamic Head (DyHead) detector head with an attention mechanism; unnecessary background information around the pedestrian object is also effectively excluded, making the model learn more concentrated feature representations. Results: Compared with the original model, the log-average miss rate of the improved YOLOv7 model is significantly reduced in both the Citypersons dataset and the INRIA dataset. Conclusions: The improved YOLOv7 model proposed in this paper achieved good performance improvement in different pedestrian detection problems. The research in this paper has important reference significance for pedestrian detection in complex scenes such as small, occluded and overlapping objects.

## 1. Introduction

Pedestrian detection is an important research field in the direction of computer vision, and deep learning has a wide range of application prospects in the field of pedestrian detection, involving many different application scenarios, such as traffic safety, intelligent driving, security and monitoring systems, intelligent robotics, drone monitoring and rescue, etc.

In the research and development of object detection, a total of two key periods have been experienced. The first is the exploration period of traditional object detection methods, and the other is the period of rapid development of object detection algorithms based on deep learning. Before 2014, the mainstream algorithm in the field of object detection was still the traditional object detection algorithm, and the implementation of the traditional object detection algorithm mainly consisted of the following three steps: Firstly, the various regions of the image are exhaustively searched through sliding windows and other methods. Then, the feature extraction algorithms such as Histograms of Oriented Gradients (HOG) [1] and Scale-Invariant Feature Transform (SIFT) [2] are adopted to extract the features of the object region, obtaining the features of the object region. Finally, the extracted features of the object region are input into the classification algorithms such as Support Vector Machine (SVM) [3] and Adaboost [4] to obtain the classification results. These traditional object detection algorithms are designed manually to extract the features of the object region, and the extracted features directly affect the detection results. Therefore, there are relatively high requirements for the debugging experience of the debugger. Compared with the object detection algorithms based on deep learning, the feature extraction of traditional object detection algorithms is relatively complex, and it is difficult to extract deeper features. Nevertheless, the ideas of sliding window, difficult sample mining and bounding box regression used in traditional object detection algorithms still affect the subsequent development of deep learning-based object detection algorithms.

With the deepening of subsequent convolutional neural network research, object detection methods based on deep learning have gradually become mainstream. The object detection methods based on deep learning are mainly divided into two main categories. One is the Two-Stage model, whose main idea is to divide the object detection task into two stages: region proposal generation and in-box object classification, and the most representative algorithm is the Regions with CNN features (R-CNN) [5,6,7] series algorithm. R-CNN [5] is the first algorithm that successfully applies deep learning to object detection, which replaces the process of manually extracting features in traditional object detection algorithms with the use of CNN to extract features, thus improving the detection accuracy. Due to the slow speed of the traditional candidate box search algorithm in R-CNN in exhaustively searching for candidate regions, Girshick et al. proposed Fast R-CNN [6] and Faster R-CNN [7] for different improvements to reduce computational redundancy. However, it still cannot match real-time detection in terms of detection speed. The other category is the One-Stage model, whose main idea is to consider the object detection task as a model that predicts the bounding box and category problems directly from the image. The One-Stage algorithms are represented by the Single-Shot Multibox Detector (SSD) [8] and You Only Look Once (YOLO) [9] series algorithms, which use a single convolutional neural network to directly predict the probability of the object bounding box and its category, transforming the object detection problem into a regression problem. Compared with the Two-Stage algorithms, the YOLO series of algorithms achieves a significant improvement in terms of detection accuracy and speed, which is able to satisfy the basic needs of pedestrian detection.

Among the numerous tasks of object detection, pedestrians are characterized by occupying a small image area, diverse postures, complex scenes and severe occlusion in the object detection task, which brings significant challenges to the accurate detection of pedestrian objects. In recent years, many experts and scholars have achieved desirable results in the field of pedestrian detection. For example, Fan Tang et al. [10] proposed TOD-YOLOv7, a small object detection method based on YOLOv7; the YOLOv7 network was reconstructed to increase the small object detection layer, and then the gated convolution module was utilized to realize the interaction with the higher-order space. In addition, coordinate attention was added to the feature extraction network of YOLOv7 to enhance the pedestrian object information, which effectively strengthened the detection ability of the network to detect pedestrians with small objects. Huanhuan Wang et al. [11] proposed a detector–tracker integration framework for pedestrian tracking in self-driving vehicles, established a pedestrian object detector based on an improved YOLOv7 network, improved the YOLOv7 backbone network with Space-to-Depth convolution, and also proposed a new appearance feature extraction network. The framework proposed in this article has good robustness for pedestrian detection in various climatic environments. Chunluan Zhou et al. [12] proposed the BiBox pedestrian detection framework, which employs a part-based pedestrian detection method that utilizes a network to simultaneously perform overall pedestrian detection and occlusion estimation. The main idea is to reduce the omission rate of occluded pedestrians and the false detection rate of unoccluded pedestrians by weighting the global features of the overall pedestrians with the local features of the pedestrian parts. Wang et al. [13] proposed the Repulsion Loss Function, which significantly improves the algorithm’s ability to detect occluded objects by moving candidate boxes closer to the matching real object box, keeping them away from other real object boxes around the corresponding real object box, and also keeping them away from candidate boxes of other real object boxes. Repulsion Loss greatly improves the detection ability of the algorithm for occluded objects by improving the regression method in the object detection algorithm.

The numerous experts and scholars mentioned above have conducted corresponding research in the field of pedestrian detection to address various difficulties that pedestrian detection faces. In the practical application of pedestrian detection, most of the tasks require high real-time performance of the model, and in the actual deployment process, the number of parameters and the amount of computation are also important factors to be considered. In this way, the One-Stage object detection network YOLOv7 [14] model is selected to improve the accuracy of the model under the condition of satisfying real-time performance.

In this study, CBAM [15] and DCNv2 [16] are introduced into two of the ELAN modules of the YOLOv7 backbone network, which effectively improves the feature extraction capability of the model and the modeling capability for geometric transformations, and also replaces the DyHead [17] with the attention mechanism to improve the information mining capability of the model for visual context. The detection head with attention mechanism is also replaced to improve the information mining ability of the model for visual context, which improves the accuracy of the model while reducing the number of parameters and computation amount, and reduces the missed detection rate and false detection rate of the model.

In response to the characteristics of pedestrian objects with various postures, different sizes and severe occlusion, we conducted experiments by using the Citypersons dataset with more occluded pedestrian objects [18] and the INRIA dataset [1], which has significant differences in pedestrian morphology and size. The experimental results show that the improved YOLOv7 model in this paper produces significant comprehensive performance improvement on these two pedestrian datasets with significant differences in style. By dividing the pedestrian objects in these two datasets into different subsets, the effectiveness and generalization of the improved YOLOv7 model in this paper for the detection of pedestrians with small, occluded and overlapping objects are fully verified.

In summary, this paper makes the following contributions:

(1) The CBAM is used after channel splicing in the two feature extraction ELAN modules of the backbone feature extraction network of YOLOv7, which enhances the model’s ability to extract features in the two different dimensions of channel and space, as well as improves the model’s perception of meaningful information in the input image.

(2) The DCNv2 is introduced into the two feature extraction ELAN modules of the backbone feature extraction network of YOLOv7, and the 3 × 3 convolution of the ELAN module is replaced by deformable convolution DCNv2 for the characteristics of diverse postures and complex scenes of the pedestrian objects, which improves the modeling ability of the model for geometric transformations, and also efficiently eliminates the redundant contextual information around the pedestrian objects, enabling the model to learn more centralized feature representations.

(3) The detection head of YOLOv7 is replaced with a DyHead with attention mechanism, which unifies scale perception, spatial perception and task perception, enabling the model to effectively adapt to objects of different scales and complexities, and improving its performance in multi-scale scenarios.

(4) By improving the YOLOv7 model, the detection ability of the model for small targets, occluded targets and crowded and overlapping pedestrian targets is effectively enhanced, providing a detection method that is more computationally efficient and has fewer parameters, higher accuracy and better generalization for the field of pedestrian target detection.

## 2. Dataset Description

**Figure 1:**
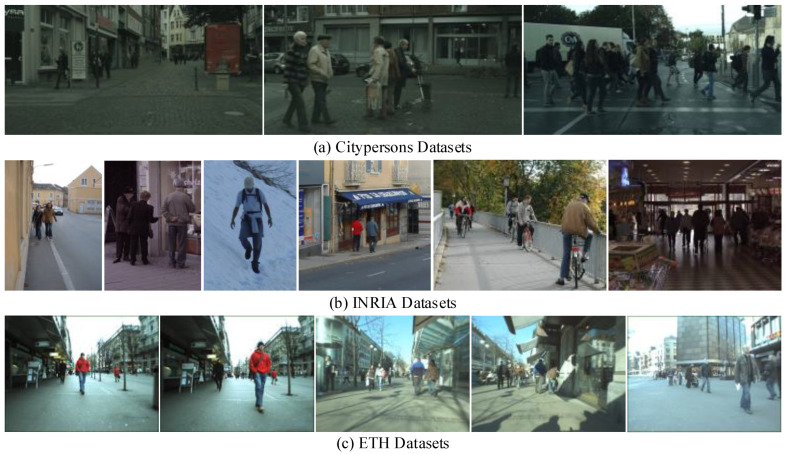
Diagram of overall situation dataset. (**a**–**c**) are partial images from the Citypersons, INRIA, and ETH datasets, respectively.

The datasets used in this paper are Citypersons [18], INRIA [1] and ETH [19]. The experimental part of this paper describes ablation experiments on the improved model and comparison experiments with other generalized object detection models in the Citypersons dataset. The final improved model is compared with other existing pedestrian detection models in the INRIA dataset. The generalization of the improved YOLOv7 model on the ETH dataset is verified to fully demonstrate the effectiveness and generalization of the improved model in improving pedestrian object detection performance. The overall dataset used in this paper is shown in Figure 1.

### 2.1. CityPersons Dataset

The Citypersons dataset [18] is a subset of the Cityscapes dataset that is only annotated for pedestrians. The dataset contains images of street scenes taken in 27 different cities, including 2975 images for training, 500 images for verification and 1575 images for testing, with a resolution of 2048 × 1024 pixels. This paper uses the training set and validation set of the Citypersons dataset for training and testing, in which the training set has 2500 positive samples and 475 negative samples, and the validation set has 441 positive samples and 59 negative samples. Since this dataset contains more small objects and severely occluded pedestrian objects, this paper pays more attention to the detection capability of the validation model for the small objects and severely occluded pedestrian objects when validating with the Citypersons dataset.

### 2.2. INRIA Dataset

The INRIA dataset [1] was collected by Navneet Dalal in his research on detecting upright pedestrians in images and videos. There are a total of 2573 pedestrian images with different resolutions in this dataset, including 614 positive samples and 1218 negative samples in the training set and 288 positive samples and 453 negative samples in the testing set. Most of the human bodies in the images of the dataset have a standing posture and a height more than 100 pixels. In this paper, a complete training set containing 1832 images with positive and negative samples was used for training. In order to ensure that the detection indicators of other pedestrian detection models are evaluated under the same conditions, only 288 images of the positive sample test set were used for the evaluation of model detection results during testing. Due to the fact that the labeled bounding boxes have a very large difference in width, the height of all bounding boxes was standardized to 0.41 times the width in the evaluation. This paper pays more attention to the recognition ability of the model to pedestrian objects with different overlap degrees when verifying with the INRIA dataset.

### 2.3. ETH Dataset

The ETH dataset [19] was captured by an AVT Marlins F033C vehicle-mounted camera installed on a car, containing a total of 1804 images. The dataset was captured in three different scenes, which had 999, 451 and 354 images, respectively, with an image resolution of 640 × 480 pixels and a frame rate of 13–14 FPS. The images were captured in the city center, which contains a large number of crowded pedestrians. Since the dataset as a whole was captured by frame extraction from the video stream, it contains a large number of consecutive pedestrian objects of different sizes.

The division of the training and test sets in the constructed dataset follows the original division criteria set by the dataset’s publisher. The overall situation of the dataset constructed in this paper is shown in Table 1.

## 3. Construction of Pedestrian Detection Network

### 3.1. Principle of YOLOv7 Model

**Figure 2:**
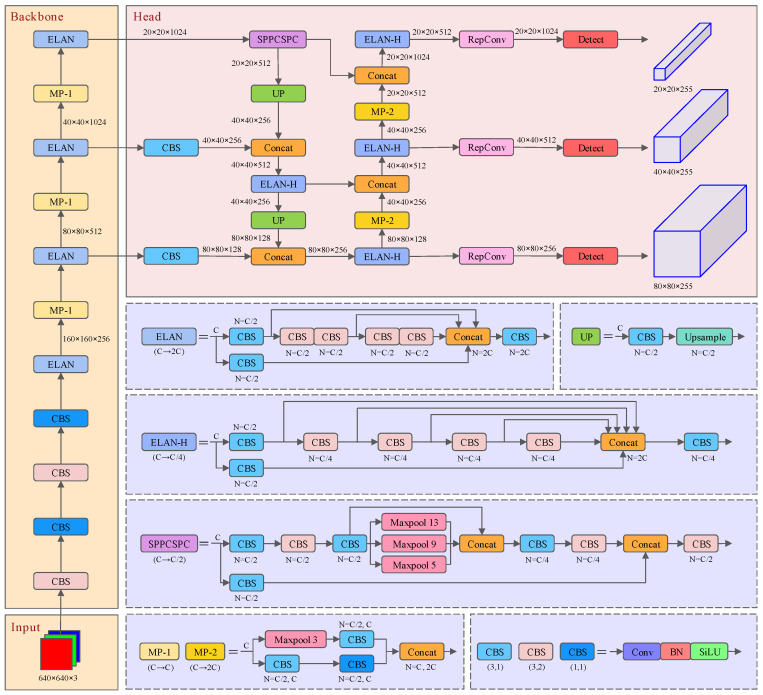
Structure diagram of the YOLOv7 network model.

The core idea of the YOLO series of algorithms is to transform the task of object detection into a regression problem and simultaneously localize and classify the objects via a single neural network to achieve real-time and efficient object detection. The YOLO series of algorithms stands out because of its remarkable balance between speed and accuracy, as well as its simplicity of structure and convenience of deployment, which makes it a widely used algorithm in practical engineering applications. Within the range of 5FPS to 160 FPS, YOLOv7 outperforms all previous object detectors in terms of speed and accuracy. Compared with YOLOv7, YOLOv8 [20] has improved accuracy, which is mainly due to its optimized network structure and innovative points. However, despite the improved accuracy of YOLOv8, YOLOv7 has a lower *MR*^−2^ than YOLOv8 in terms of missed detection and false detection for pedestrian object detection. In the actual application scenarios of pedestrian detection tasks, there are higher requirements for reducing missed detection and false detection in most cases. In this case, YOLOv7 is selected as the baseline of the improved model in this paper. The structure of the YOLOv7 network model is shown in Figure 2.

Compared to previous YOLO series network models, the main improvements and modules of YOLOv7 include the following:

(1) SPPCSPC module

The YOLOv7 model uses the SPPCSPC module at the backbone and head docking, which is mainly composed of SPP [21] and CSPNet [22]. The core idea of the SPP structure is to obtain different scale and size features of the input feature layer through maximum pooling operations of different sizes, and integrate them together to provide richer visual context information for image features. The CSPNet network first divides the feature maps of the base layer into two parts, and then merges them through the proposed cross-level hierarchy. The main idea is to split the gradient flow so that the gradient flow propagates through different network paths. Through this operation, the amount of calculation is effectively reduced, and the speed and accuracy of network reasoning are also improved.

(2) ELAN module

**Figure 3:**
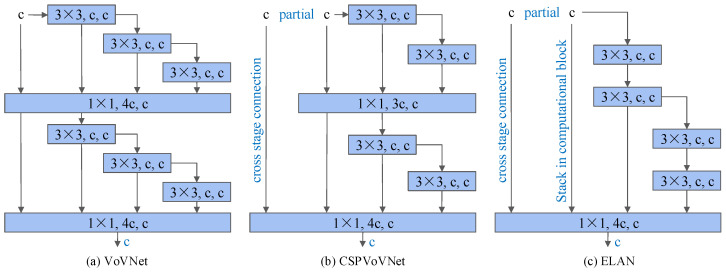
Schematic diagram of the Efficient Layer Aggregation Network (ELAN). (**a**–**c**) represent the network structures of VoVNet, CSPVoVNet, and ELAN, respectively.

YOLOv7 uses the ELAN module in the feature extraction network part, which enables the network to learn more features and be more robust by controlling the shortest and longest gradient paths. This is a layer aggregation architecture with efficient gradient propagation paths, which solves the problem that the convergence of the deep model will gradually deteriorate when performing model scaling. The ELAN module is mainly made up of VoVNet [23] and CSPNet [22]. This module avoids the use of too many transition layers by using a stack structure in the computational block, which makes the gradient path of the entire network the shortest, and thus allows the length of the network to rapidly become longer and deeper to learn and converge effectively as well. The structure of the ELAN module is shown in the Figure 3.

(3) Auxiliary training module

The YOLOv7 model uses a deeply supervised training method to train the network in the middle layer of the network. This training method assists network training by adding additional auxiliary heads in the middle layer of the network and using the loss of the auxiliary heads as a guide. Compared with the traditional end-to-end training method, the deeply supervised training method can better propagate the gradient through the supervised signals in the middle layer, which helps to alleviate the problem of gradient disappearance. In addition, deep supervision can also guide the network to learn more generalized features faster, not only for the final task, but also effectively improves the generalization ability of the network.

**Figure 4:**
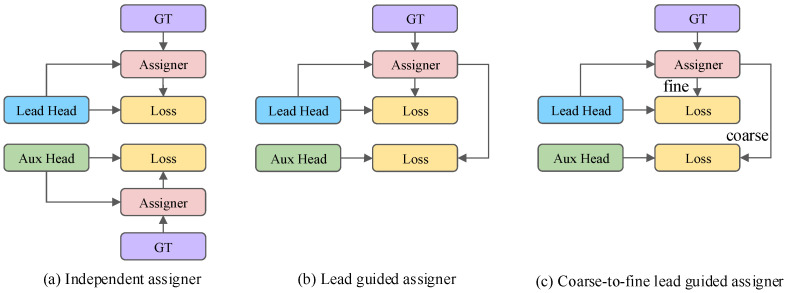
Coarse for auxiliary and fine for lead head label assigner. (**a**–**c**) represent three different label assignment methods, respectively.

In terms of the issue of label assignment, the traditional label assignment method is to separate the lead head and auxiliary head, and assign labels based on their respective prediction results and ground truth (as shown in Figure 4a). In YOLOv7, the authors generate soft labels based on the prediction results of the lead head and the calculation of ground truth, and then use the generated soft labels to guide the training of the auxiliary head and lead head. The specific deep supervised label assignment strategy is shown in Figure 4.

Lead head guided label assigner: The guided label assigner calculates the soft label based on the prediction result and ground truth of the lead head, and then uses this set of generated soft labels to guide the model training of the auxiliary head and the lead head. Due to the stronger learning ability of the lead head compared with the auxiliary head, the soft labels generated by the lead head can better represent the distribution and correlation between the source data and the object data. Therefore, the shallow auxiliary head can be used to directly learn the information learned by the lead head, so that the lead head can pay more attention to learning the residual information that has not been learned.

Coarse-to-fine lead head guided label assigner: The coarse-to-fine lead head obtains two different sets of soft labels, namely coarse labels and fine labels, based on the prediction results of the lead head and the calculation of the ground truth. The coarse labels serve the purpose of reducing the constraints on the allocation of positive samples so as to allow more grids to be considered as positive objects, while the fine labels serve the same purpose as the soft labels generated by the guidance label allocator of the lead head, which is used to guide the training of the auxiliary head and lead head. Due to the inferior learning ability of the auxiliary head compared to the lead head, the recall rate of the auxiliary head is optimized to avoid the loss of important information. For the output of the lead head, the results with higher accuracy are selected from the higher recall results as the final output.

### 3.2. Improvement of YOLOv7 Model

**Figure 5:**
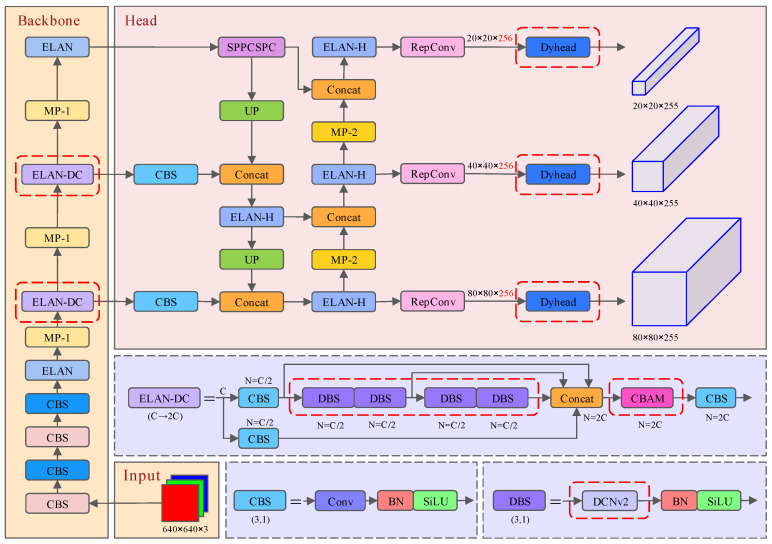
Diagram of improved YOLOv7 network structure.

The improvement of the YOLOv7 model is mainly reflected in three aspects, as shown in Figure 5.

(1) The CBAM is used after channel concatenation between the two feature extraction ELAN modules of the backbone feature extraction network [15]. The attention mechanism strengthens the feature extraction ability of the model in the two different dimensions of channel and space by sequentially applying the channel attention module and the spatial attention module, so that the model can better learn the object information and the position information in the channel and space, enhancing the model’s perception of the meaningful information in the input image and improving the network’s recognition ability of the important features in the complex images, thereby improving the accuracy and generalization ability of the model.

(2) The 3 × 3 convolution of the two feature extraction ELAN modules of the backbone feature extraction network is replaced by DCNv2 [16]. DCNv2 has better modeling ability for geometric transformation compared with the standard convolutional neural network. For the pedestrian object detection task, due to the characteristics of pedestrian objects with diverse postures and complex scenes, a good modeling ability for geometric transformation can better express the characteristics of the diverse features of pedestrian objects. The offset of the convolutional layer sampling points and the modulation scalar of the modulated deformable RoI pooling module are reformulated by controlling the amplitude of the learned features in DCNv2, and the network is carried out by R-CNN feature imitation, which effectively eliminates the redundant contextual information so that the model learns a more centralized representation of the features, and effectively solves the problem of complex scenes in pedestrian object detection tasks.

(3) The detection head of YOLOv7 is replaced with the DyHead detection head with an attention mechanism [17], which allows the network to dynamically adjust the attention head in the network at different layers and scales, enabling the model to effectively adapt to objects of different scales and complexities, and improving the performance of the model in multi-scale scenarios. Moreover, the detection head also integrates scale perception, spatial perception and task perception, which significantly improves the perception ability of the object detection head towards pedestrian objects.

### 3.3. Principle of Adding CBAM

**Figure 6:**
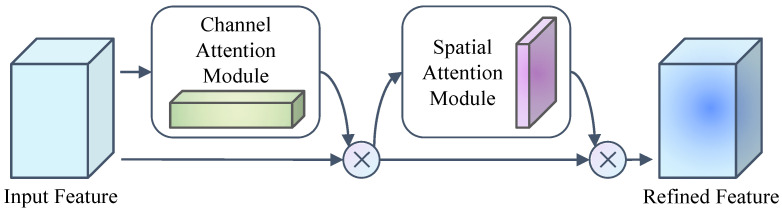
Convolutional Block Attention Module (CBAM).

The data features are extracted by mixing the cross-channel and spatial information during the process of convolutional operation. In order to extract more meaningful features in each dimension, this paper employs the CBAM [15] module to enhance the meaningful features under the two dimensions of channel and space. To this end, the CBAM module applies the channel and spatial attention modules sequentially so that each branch can learn the object information and position information in the channel and space, respectively. Therefore, this paper effectively improves data flow in the network by using the CBAM module to learn significant features and suppress unimportant features. The structure of the Convolutional Block Attention Module network is shown in Figure 6.

The CBAM module is a simple but effective feed-forward convolutional neural network attention module. The module contains two independent sub-modules, namely a channel attention module and a spatial attention module, which receive the output result *F* ∈ *R*^*C*×*H*×*W*^ of an intermediate convolutional layer as feature data input. The intermediate feature data are obtained by sequentially obtaining a channel attention feature map *M_c_* ∈ *R*^*C*×1×1^ and a spatial attention feature map *M_s_* ∈ *R*^1×*H*×*W*^ under two different dimensions; that is, it passes through a channel attention module first, and after the weighting calculation of this channel attention module, it will be further weighted by a spatial attention module. In order to make the feature output more refined each time in different dimensions, the attention feature map after each attention weighting computation will be multiplied by the input feature map for adaptive feature refinement, and the final output result will be obtained. The process can be expressed as:

(1) $$\begin{array}{l} F^{\prime }=M_{c}(F)⊗F\\ F^{\prime \prime }=M_{s}(F^{\prime })⊗F^{\prime } \end{array}$$

wherein ⨂ denotes element-wise multiplication.

**Figure 7:**
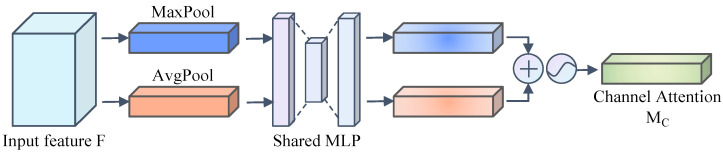
Channel attention module.

Channel attention module: The sub-module first aggregates the spatial information of the input feature maps by performing maximum pooling and average pooling operations on the input feature maps, respectively, so as to obtain the maximum pooling feature $F_{max}^{c}$ and the average pooling feature $F_{avg}^{c}$ of the input feature maps. Then, these two pooled features obtained through different operations are fed into a multi-layer perceptron shared network with a hidden layer. After passing through the shared network, these two pooled features obtain a channel attention feature map *M_c_* ∈ *R*^*C*×1×1^. Finally, these two output feature vectors processed by the shared network are merged using element-by-element summation. The structure of the channel attention module is shown in Figure 7. The calculation formula is as follows:

(2) $$\begin{array}{l} M_{c}(F)=\sigma (MLP(AvgPool(F))+MLP(MaxPool(F)))\\ =\sigma (W_{1}(W_{0}(F_{avg}^{c}))+W_{1}(W_{0}(F_{max}^{c}))) \end{array}$$

wherein *σ* denotes the sigmoid function, and *W*_0_ and *W*_1_ denote the *MLP* weights shared by the two inputs.

**Figure 8:**
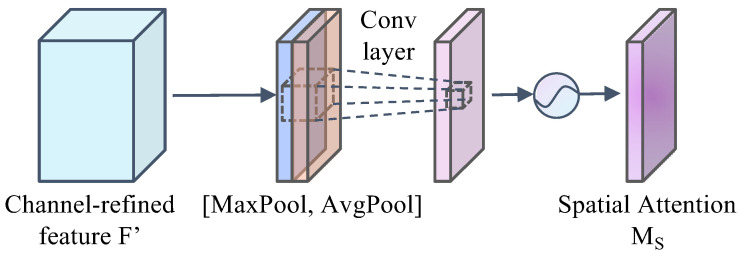
Spatial attention module.

Spatial attention module: Unlike channel attention, spatial attention focuses on location information and is complementary to channel attention. The principle of this sub-module is to take the feature map output from the channel attention module as the input. Firstly, the input feature map is subjected to maximum pooling and average pooling operations to aggregate the channel information of the feature map, and the maximum pooling feature $F_{max}^{s}$ ∈ *R*^1×*H*×*W*^ and the average pooling feature $F_{avg}^{s}$ ∈ *R*^1×*H*×*W*^ of the input feature map are obtained, and then the two pooling features are subjected to channel splicing operations based on the channels. Then, a 7 × 7 convolutional layer and sigmoid activation function are used to generate the spatial attention feature map *M_s_* ∈ *R*^*H*×*W*^. The structure of the spatial attention module is shown in Figure 8. The spatial attention is calculated as follows:

(3) $$\begin{array}{l} M_{s}(F)=\sigma (f^{7\times 7}([AvgPool(F);MaxPool(F)]))\\ =\sigma (f^{7\times 7}([F_{avg}^{s};F_{max}^{s}])) \end{array}$$

wherein *f*^7×7^ denotes a convolution operation with a convolution kernel size of 7 × 7.

### 3.4. Principle of Replacing ConvNets with DCNv2

Due to the fixed geometric structure in the module constructed by the convolutional neural network, its modeling ability for geometric transformations is very limited. Therefore, the deformable convolution module and the deformable RoI pooling module are introduced in the DCNv1 [24] to enhance the modeling ability of convolutional neural networks for geometric transformations. The core idea of both modules introduced in DCNv1 is add additional offset based on the spatial sampling position information in the module and learn this offset from the object task.

DCNv1 has significantly enhanced the modelling ability of geometric transformations by introducing deformable convolutions. However, some scholars have found that although its feature space support is more in line with the object structure of the object compared to the standard convolution, this spatial support still has a large portion of the region beyond the desired region of interest, and thus DCNv2 was proposed [16]. DCNv2 introduces a modulation mechanism based on DCNv1, so that the deformable convolution module can not only learn the magnitude of the offset, but also modulate it by learning the feature magnitude in order to realize the change of the sample spatial distribution and relative influence. In addition, DCNv2 proposes a feature simulation scheme to guide the network training, which further improves the modeling capability of the deformable convolution module.

**Figure 9:**
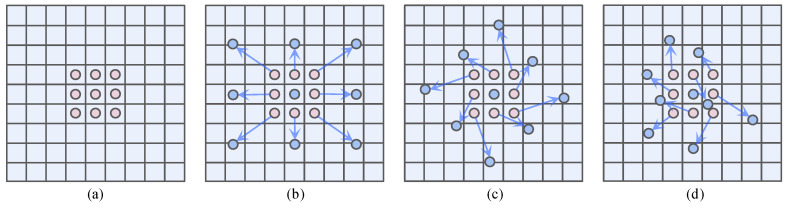
Sampling positions in 3 × 3 standard and deformable convolutions. (**a**) standard convolution sampling method; (**b**,**c**) denotes the special transformation case where deformable convolution generalizes the offset, offset direction and rotation; (**d**) general sampling method for deformable convolution.

Figure 9 shows the sampling positions of standard convolution and deformable convolution.

The principle of deformable convolution is as follows: For the implementation process of standard convolution, given a convolution kernel of *K* sampling positions, the input feature map *x* is first sampled and then the sampled values weighted by *w* are summed. For each position *p* on the output feature map *y*, the formula is expressed as follows:

(4) $$y(p)=\sum_{k=1}^{K}w_{k}\cdot x(p+p_{k})$$

wherein *p_k_* denotes the pre-specified offset at the *k*-th position of the convolution kernel, and for a 3 × 3 standard convolution kernel with *N* = 9, *p_k_* ∈ {(−1, −1), (−1, 0), …, (0, 1), (1, 1)}.

In the deformable convolution, a learnable parameter ∆*p_k_* is added to the scope of the convolution operation, which denotes the learnable offset of the *k*-th sampling position in the deformable convolution. As a consequence, Equation (4) can be changed to:

(5) $$y(p)=\sum_{k=1}^{K}w_{k}\cdot x(p+p_{k}+\Delta p_{k})$$

Since offsets are usually non-integer, Equation (5) is realized by bilinear interpolation as follows:

(6) $$x(p)=\sum_{q}G(q,p)\cdot x(q)$$

wherein *p* denotes an arbitrary sampling position, *q* represents all integer space positions in the enumeration feature map *x*, and *G* is a bilinear interpolation kernel function, which can be divided into two one-dimensional kernels:

(7) $$G(q,p)=g(q_{x},p_{x})\cdot g(q_{y},p_{y})$$

wherein *g*(*a*, *b*) = max(0, 1 − |*a* − *b*|).

**Figure 10:**
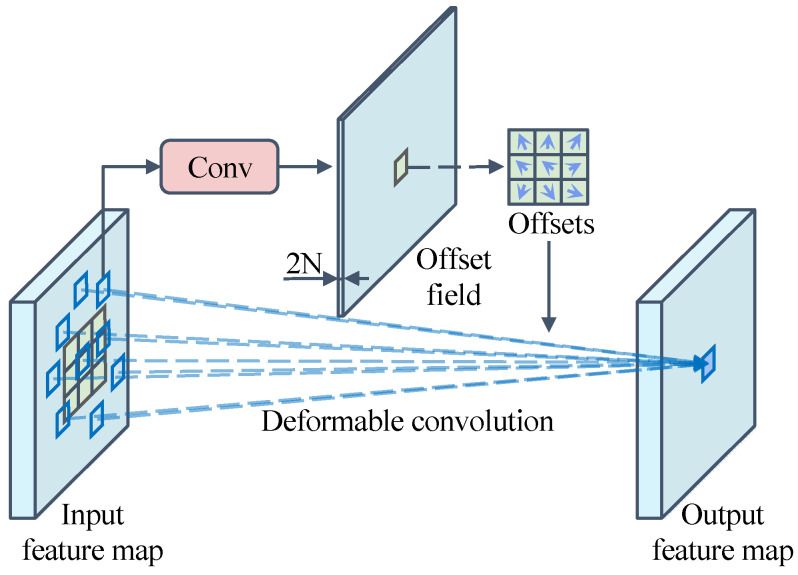
Schematic diagram of the implementation principle of 3 × 3 deformable convolution.

The acquisition of offset is shown in Figure 10. For a 3 × 3 deformable convolution, the offset output required for deformable convolution is first obtained by applying a convolution layer on the same input feature map (the number of offset field channels of the output is 2*N*, which represents the offsets in the *x* and *y* directions), and then the obtained offset is applied to the convolution kernel to achieve the deformable convolution effect.

The principle of deformable RoI pooling is as follows: The RoI pooling module can convert an input rectangular region of arbitrary size into a fixed-size feature, which is realized as follows: given a RoI of size *w* × *h*, RoI pooling divides the RoI into *m* × *m* blocks and finally obtains an output feature map *y* with a size of *m* × *m* through pooling operation. The pooling operation for the *k*-th block is defined as:

(8) $$y(k)=\sum_{j=1}^{n_{k}}x(p_{kj})/n_{k}$$

wherein *p_kj_* denotes the sampling location of the *j*-th network unit in the k-th block and *n_k_* denotes the number of network units within the *k*-th sampling block.

Similarly, an offset ∆*p_k_* is added to the deformable RoI pooling, and then Equation (8) can be changed to:

(9) $$y(k)=\sum_{j=1}^{n_{k}}x(p_{kj}+\Delta p_{k})/n_{k}$$

**Figure 11:**
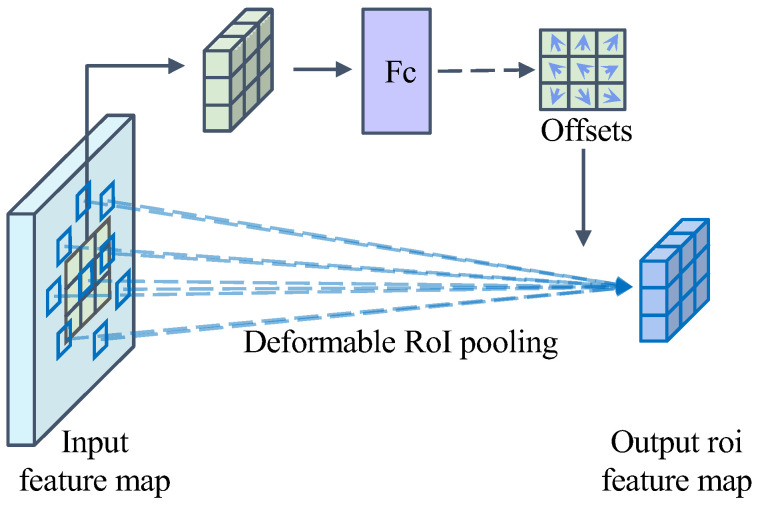
Schematic diagram of the implementation principle of 3 × 3 deformable RoI pooling.

Figure 11 illustrates how deformable RoI pooling obtains offsets. Firstly, the pooled feature maps are obtained by Equation (8), and then the normalized offset output is obtained by applying a fully connected layer over the same input feature maps, which is then converted to the final offset ∆*p_k_* by multiplying it with the width and height of the ROI.

(10) $$\Delta P_{k}=\gamma \cdot \Delta {\hat{P}}_{k}\cdot (w,h)$$

wherein *γ* is the scalar used to adjust the magnitude of the offset.

The above is the implementation principle of the DCNv1. In DCNv2, the offset is redefined by introducing learnable feature magnitudes to further improve its capability. The modulation deformable convolution can be expressed as:

(11) $$y(p)=\sum_{k=1}^{K}w_{k}\cdot x(p+p_{k}+\Delta p_{k})\cdot \Delta m_{k}$$

wherein Δ*p_k_* and Δ*m_k_* are the learnable offset and modulation scalar at the *k*-th position, respectively. The modulation scalar Δ*m_k_* is located in the range [0, 1]. Both the offset and modulation scalars are obtained by applying a convolutional layer on the same input feature mapping (the number of output offset field channels is 3*N*, the first 2*N* channels represent the offset in the *x* and *y* directions, and the remaining *N* channels are further transmitted to the sigmoid layer to obtain the modulation scalars).

The modulation deformable RoI pooling is designed similarly. Δp_k_ and Δm_k_ are regarded as the learnable offset and modulation scalar of the k-th block, and the modulation deformable RoI pooling is calculated as follows:

(12) $$y(k)=\sum_{j=1}^{n_{k}}x(p_{kj}+\Delta p_{k})\cdot \Delta m_{k}/n_{k}$$

For both the standard convolutional neural network and the deformable convolutional neural network, the redundant contextual information outside the RoI will affect the extracted features, thereby having an impact on the final detection results. For the deformable convolutional neural network DCNv2, the weight of each sampling point can be controlled by controlling the modulation scalar of the modulation deformable RoI pooling module to eliminate the redundant contextual information. In order to further reduce the interference of the redundant contextual information and improve its ability in adjusting the spatial support region, DCNv2 guides the features after RoI pooling to simulate the features of R-CNN by constructing an R-CNN teacher network. Through this auxiliary training module DCNv2, a more concentrated feature representation can be learned.

**Figure 12:**
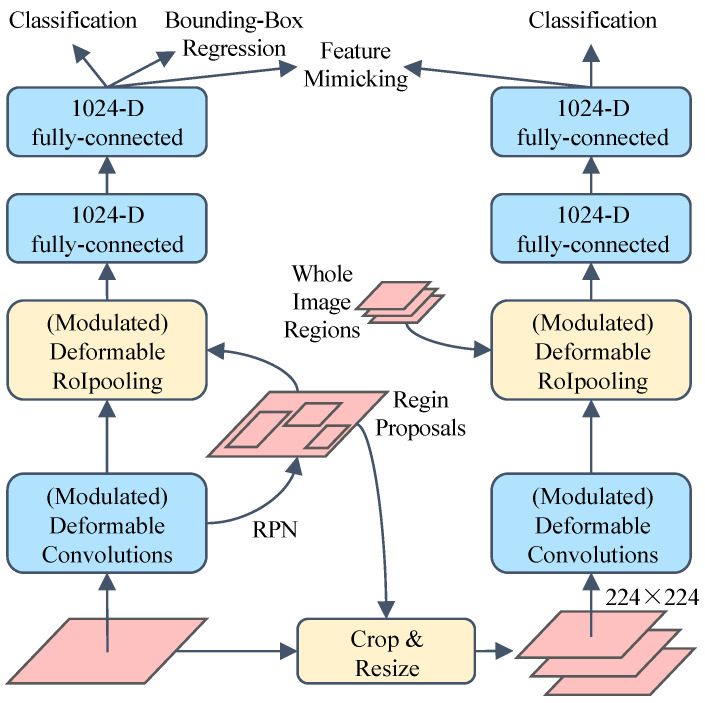
Network training using R-CNN feature imitation.

The network architecture of the deformable Faster R-CNN used for training is shown in Figure 12. Firstly, the RoI b used for feature imitation is given, and the corresponding image blocks are cropped and resized to 224 × 224 pixels. In the R-CNN branch, the backbone network operates on the resized image blocks and generates feature maps with a spatial resolution of 14 × 14. The modulated deformable RoI pooling layer is then applied to the top of the feature map. Afterwards, two fully connected layers of 1024-D are applied to generate R-CNN features of the input image block, denoted by *f_RCNN_* (*b*). Similarly, Faster R-CNN features corresponding to the R-CNN features are denoted by *f_FRCNN_* (*b*). The classification is then performed using a Softmax classifier, and the feature simulation loss is defined on the cosine similarity between *f_RCNN_* (*b*) and *f_FRCNN_* (*b*), which can be calculated as:

(13) $$L_{minic}=\sum_{b\in \Omega }\left[1-\cos(f_{RCNN}(b),f_{FRCNN}(b))\right]$$

The total loss during network training consists of the feature imitation loss and the R-CNN classification loss as well as the original loss term in the Faster R-CNN. In inference, only the Faster R-CNN network is used for image testing, so that R-CNN feature imitation does not introduce additional computation in inference.

### 3.5. Principle of Replacing Detection Head with DyHead

The object detection head is mainly used for multi-scale object detection of the features extracted from the backbone network. Firstly, in order to enable the object detection network to better detect accurate objects, the object detection head should have good scale perception ability to cope with objects of different scale sizes in the image. Secondly, the detection head should also have good spatial awareness to deal with the objects presenting different shapes, positions and orientations from different perspectives. Thirdly, the detection head should also have good task awareness, because the same object to be detected can be expressed in different ways such as bounding boxes, centers, corners, etc. Therefore, good task awareness can have faster learning ability for different expressions.

**Figure 13:**
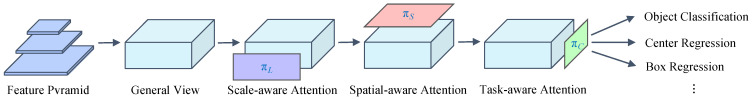
Diagram of the DyHead method.

In order to enhance the scale perception ability, spatial awareness and task awareness of the object detection head, a dynamic head framework that combines the object detection head with attention is applied in this paper. The coherent combination of multi-head self-attention mechanism within the feature layer of scale perception, the spatial location of spatial perception, and the output channel of task perception unifies scale perception, spatial perception and task perception, which significantly improves the perception ability of object detection heads towards pedestrian objects. The specific theoretical implementation of the DyHead is shown in Figure 13.

Given a characteristic tensor *F* ∈ *R*^*L*×*S*×*C*^, the general formula for applying self-attention is:

(14) W(F)=π(F)⋅F

wherein *π* is the attention function. Due to the high dimensionality of tensors, the direct calculation of the attention function for all dimensions will result in a large amount of computational cost. Therefore, the consumption of computational resources can be reduced by converting the attention function into three consecutive attentions from three different perspectives. The calculation formula is as follows:

(15) $$W(F)=\pi_{C}(\pi_{S}(\pi_{L}(F)\cdot F)\cdot F)\cdot F$$

wherein *π_L_*, *π_S_* and *π_C_* denote the attention functions applied to the three different dimensions of *L*, *S* and *C*, respectively, and the calculation formula is as follows:

Scale-aware attention *π_L_*: features of different scales are dynamically fused according to semantic importance:

(16) $$\pi_{L}(F)\cdot F=\sigma (f(\frac{1}{SC}\sum_{S,C}F))\cdot F$$

wherein *f* is a linear function approximated by a 1 × 1 convolutional layer, and *σ*(*x*) = max(0, min(1, (*x* + 1)/2)) is a hard-sigmoid function.

Spatially aware attention *π_S_*: by using deformable convolutional [16] to make attention learning sparse, and then aggregating different levels of features at the same spatial position.

(17) $$\pi_{S}(F)\cdot F=\frac{1}{L}\sum_{l=1}^{L}\sum_{k=1}^{K}w_{l,k}\cdot F(l;p_{k}+\Delta p_{k};c)\cdot \Delta m_{k}$$

wherein *K* denotes the number of sparse sampling positions, and Δ*p_k_* and Δ*m_k_* represent the offset and modulation scalar learned through deformable convolution, respectively.

Task-aware attention *π_C_*: in order to enable joint learning and generalize different representations of objects, task-aware attention is used at the end of the architecture, which can dynamically control the opening and closing of different feature channels to support different tasks:

(18) $$\pi_{C}(F)\cdot F=max(\alpha^{1}(F)\cdot F_{c}+\beta^{1}(F),\alpha^{2}(F)\cdot F_{c}+\beta^{2}(F))$$

wherein *F_c_* denotes the feature slice of the *c*-th channel, and [*α*^1^, *α*^2^, *β*^1^, *β*]^2T^ = *θ* (-) is the hyperfunction of the activation threshold of learning control.

**Figure 14:**
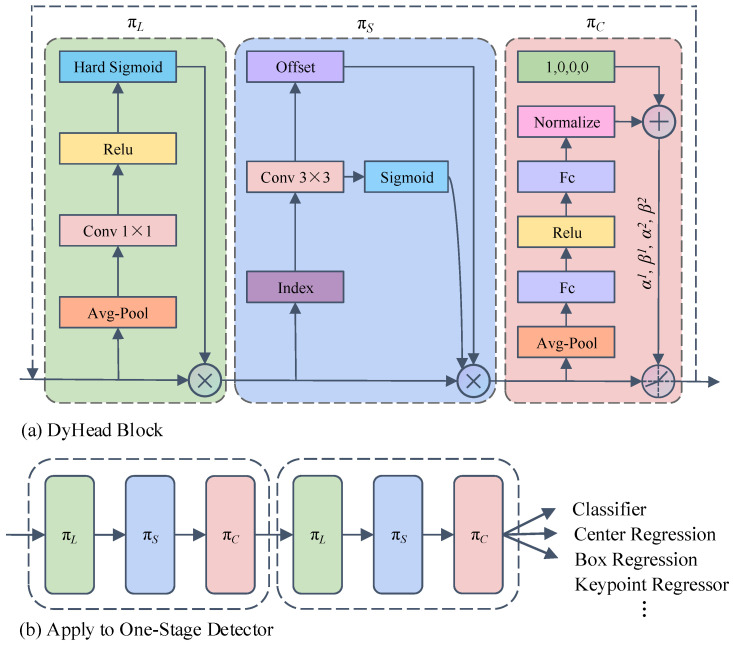
Structure diagram of the DyHead detection head.

The above three attention mechanisms can also be used to stack multiple *π_L_*, *π_S_* and *π_C_* blocks by nesting them multiple times, but each additional nesting of Equation (15) will introduce more parameter quantities and also have an impact on the inference speed and computation amount. Therefore, this paper stacks *π_L_*, *π_S_* and *π_C_* blocks by two nesting methods, which significantly improves the accuracy without increasing too much computation and parameter quantity. The detailed configuration of the DyHead block is shown in Figure 14.

## 4. Experimentation

### 4.1. Experimental Setup

The experimental platform in this paper is the Ubuntu 18.04 operating system, using the Pytorch 1.10.0 deep learning framework, with a CPU of 15 vCPU Intel(R) Xeon(R) Platinum 8358P CPU@2.60 GHz, and a GPU of RTX A5000 (24 GB), and the underlying CUDA11.3 as the parallel computing framework.

In this paper, different training strategies were developed for the Citypersons and INRIA datasets. The image size used in this paper was 2048 × 1024 pixels on the Citypersons dataset, and the Adam optimizer was selected, with the momentum set to 0.9, and Batchsize set to 24. The learning rate adjustment method was cosine annealing attenuation, with the initial learning rate set to 0.0003, and the final learning rate set to 0.2 times of the initial learning rate, and the training data was iteratively trained for 400 epochs. When using the INRIA dataset for training, the Adam optimizer was used, with momentum set to 0.9 and Batchsize set to 32. The learning rate was adjusted using cosine annealing decay, with an initial learning rate of 0.0001 and a final learning rate of 0.1 times the initial learning rate, and the training data was iteratively trained for 300 epochs. The remaining hyperparameters not specifically mentioned during model training were kept at their default values.

### 4.2. Model Evaluation Index

The commonly used evaluation indexes in object detection models are *P* (Precision), *R* (Recall), *AP* (Average Precision) and *mAP* (Mean Average Precision). *AP* is used to measure the detection precision of the model, which takes into account the balance between precision and recall under different confidence thresholds, and is an intuitive evaluation criterion for evaluating the precision of the detection model.

The calculation formula of each evaluation index is as follows:

(19) $$P=\frac{TP}{TP+FP}\times 100\%$$

(20) $$R=\frac{TP}{TP+FN}\times 100\%$$

(21) $$AP=\int_{0}^{1}P(R)dR$$

(22) $$mAP=\frac{1}{n}\sum_{i=1}^{n}AP_{i}$$

In the above evaluation index calculation formulas, *P* refers to the proportion of correct predictions among all results with positive predicted values, *R* denotes the proportion of correctly predicted results among all results with positive predicted values, and *TP* (True Positive) represents the number of positive samples of true prediction; *FP* (False Positive) represents the number of positive samples of false prediction; *FN* (False Negative) represents the number of negative samples of false prediction; *AP_i_* represents the average precision of the *i*-th object category.

In the pedestrian detection task, the commonly used evaluation indexes are *FPPI* (False Positive Per Image), *MR* (Miss Rate) and *MR*^−2^ (Log Average Miss Rate). Among them, *FPPI* represents the average false detection rate of the image. When testing the dataset, a batch of test set GT (Ground Truth) is relatively small, and a certain image will have a lot of false detection, but this batch of data has a lot of data without GT, which will lead to the mAP is relatively small and does not meet the practical requirements. In the dataset of pedestrian detection, there is a larger portion of the image does not contain pedestrians, so using the *FPPI* as an evaluation index would be more appropriate. The *MR*-*FPPI* curve can well reflect the overall performance of the detector, and *MR* and *FPPI* are two mutually exclusive indicators. When the model detection threshold is lower, the model detects more objects, less missed detection but higher false detection. When the threshold increases, the false detection decreases and the missed detection increases. In the pedestrian detection, the upper limit of the *FPPI* of each image is related to the density of pedestrians. Therefore, it is more reasonable to use the *MR*-*FPPI* curve than the Precious–Recall curve in the field of pedestrian detection. The *MR*-*FPPI* curve can be obtained by setting different detection thresholds. *MR*^−2^ is an evaluation index used to quantify the *MR*-*FPPI* curve, which is calculated by taking the *FPPI* as the horizontal coordinate, and taking *log* (*MR*) as the vertical coordinate. Nine *FPPI* values are uniformly selected within the range of [0.01, 1] to obtain the corresponding 9 *log* (*MR*) values on the *MR*-*FPPI* curve and calculate their average. Finally, the above average value is restored to the percentage form of *MR* through the exponential operation. The smaller the value of *MR*^−2^, the better the performance of the model. The calculation formula is as follows:

(23) MR=1−R

(24) $$FPPI=\frac{FP}{N}$$

(25) $$MR^{-2}=e^{\frac{1}{9}\sum \delta (FPPI)}$$

In the above evaluation index calculation formulas, *MR* is the missed detection rate, *FPPI* is the average false detection rate of the image, *N* is the number of images and *δ*(*FPPI*) denotes the mapping relationship between *FPPI* and *log* (*MR*).

When performing the model evaluation, this paper follows the criteria mentioned in Reference [25]. Due to the overall difference between the Citypersons dataset and the INRIA dataset, this paper selected a different subset partition methods when using these two datasets for model evaluation. And *AP* and *MR*^−2^ were selected as the evaluation indexes on the Citypersons dataset in this paper, and the improvement of the model was verified during ablation experiments to improve the accuracy of pedestrian object detection and the overall performance changes of false and missed detections. Additionally, the Citypersons dataset contains more small objects and severely occluded pedestrian objects. Therefore, according to the degree of pedestrian occlusion and the height of the pixels occupied by the pedestrians, the pedestrian objects are further categorized into reasonable setting, small setting and heavy occlusion in the ablation experiments when evaluating the model using the Citypersons dataset. On the INRIA dataset, due to the fact that the majority of the human bodies in the dataset is greater than 100 pixels and the specific values of the visible boxes occluding the pedestrians are not provided, only *MR*^−2^ is selected as the evaluation index when using this dataset. In addition, when the data set is divided into subsets, the evaluation of severely occluded objects and small objects is not considered. When the model evaluation of INRIA dataset is carried out, the effect of overlap on pedestrian detection is considered. Therefore, the pedestrian objects are divided into four subsets according to the overlap ratios of 0, 0.25, 0.50 and 0.75 in the model evaluation of this dataset, so as to verify the false or missed detections of the model when identifying objects with different degrees of overlap. It should be noted that the way of dividing subsets in this paper is to divide subsets through specific GT boxes. The same pedestrian target or multiple pedestrian targets in the same image can be either small targets or occluded or overlapping targets, depending on whether the GT box meets the conditions in Table 2. It is not the traditional way of dividing a single image into a specific subset. The specific data division of pedestrian objects based on different degree of occlusion, degree of overlap and occupied pixel height is shown in Table 2.

### 4.3. Ablation Experiments

In this paper, the significance of each module added during the improvement process of YOLOv7 model is verified by ablation experiments. On the basis of the YOLOv7 model, the CBAM, DCNv2 and DyHead modules are applied to test the final effect of the model. After testing, the application of CBAM, DCNv2 and DyHead on the basis of the original model significantly improved the accuracy of the model and reduced the performance of false detection and missed detection of the model. Although the *FPS* decreased to a certain extent, it can still far meet the requirements of real-time detection for pedestrian target detection tasks. Compared with the original YOLOv7 model, the *AP* value of the improved model of the final scheme increased by 3.6% (The higher the better), the total *MR*^−2^ was reduced by 3.03% (The lower the better), and the reasonable subset of the object *MR*^−2^ was reduced by 3.56%. This paper also evaluates the performance of small objects and occluded objects in pedestrian detection. In the detection of small-object pedestrians and occluded pedestrians, the values of *MR*^−2^ were reduced by 6.81% and 3.38%, respectively. The experimental results show that the performance of the improved model for detecting small-object pedestrians and occluded pedestrians is greatly improved, in which the improvement of the performance of detecting small-object pedestrians is especially obvious. The detailed information is provided in Table 3.

### 4.4. Comparative Experiments

In order to verify the comprehensive performance of the improved YOLOv7 pedestrian detection network, this paper compares the comprehensive performance of the improved YOLOv7 pedestrian detection network model with the generalized object detection models such as Faster-RCNN (Resnet50 + FPN), SSD (vgg512), YOLOv5 [26], YOLOv7 and YOLOv8 in the Citypersons dataset.

**Figure 15:**
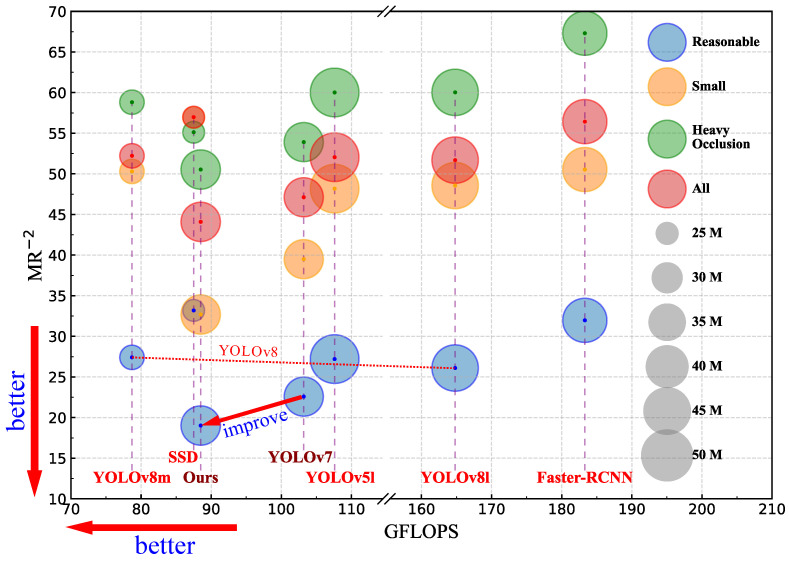
Comprehensive comparison of parameters, *GFLOPS* and *MR*^−2^.

As can be seen from the results of the comparison experiments in Table 4, the improved YOLOv7 pedestrian detection network model in this paper is 12.34% lower than the *MR*^−2^ of the Two-Stage object detection network Faster-RCNN (Resnet50 + FPN) model in terms of comprehensive performance, and the values of *MR*^−2^ are reduced by 17.84% and 16.78% on the subsets of small-object pedestrians as well as occluded-object pedestrians, respectively. The values of *MR*^−2^ are 12.89%, 7.96%, and 7.6% lower than those of the same One-Stage stage algorithm models SSD (vgg512), YOLOv5l and YOLOv8. On subsets of small-object pedestrians, the values of *MR*^−2^ are reduced by 24.25%, 15.49% and 15.87%, respectively, and on subsets of occluded-object pedestrians, the values of *MR*^−2^ are reduced by 4.59%, 9.49% and 9.5%, respectively. This paper also carried out comprehensive analysis of the number of parameters, the amount of calculation and *MR*^−2^, as shown in Figure 15, The horizontal axis of this graph represents the *GFLOPS*, and the vertical axis represents *MR*^−2^. Their values are better when smaller. Therefore, in the graph, the closer it is to the lower left corner of the coordinate axis, the better the performance. The size of the bubbles in the graph represents the number of parameters. Different colors represent the pedestrian target detection performance under different subsets. The smaller the bubble, the better. Bubbles with the same horizontal axis coordinate represent the detection results of the same model under different subsets. The same model is labeled with a label at the bottom of the graph using a dotted line. According to the comparison results, it can be seen that the improved model only increases the number of parameters by 0.2 M, which is almost negligible, but the amount of calculation and *MR*^−2^ have been reduced to a large extent, and the improved YOLOv7 model has a very good performance in parameter quantity, calculation amount and comprehensive performance of *MR*^−2^.

**Figure 16:**
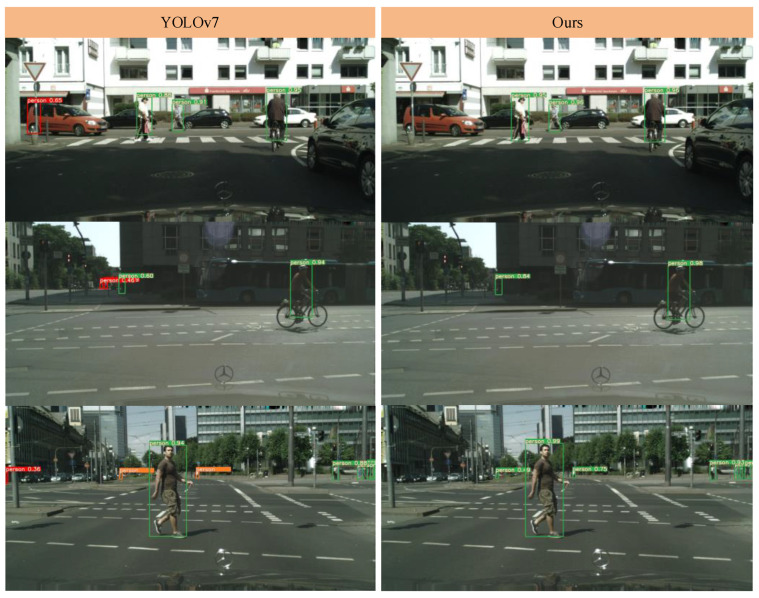
Comparison of actual detection effect.

As shown in Figure 16, it is more intuitive to observe that the accuracy of the unmodified YOLOv7 model is significantly lower than that of the improved YOLOv7 model in practical detection performance. The unmodified YOLOv7 model exhibited multiple instances of missed and incorrect detections during actual testing. In Figure 16, the green bounding boxes indicate pedestrians that were correctly detected, the red bounding boxes indicate false positives and the orange boxes indicate missed detections.

In addition, this paper compares the detection performance of the improved YOLOv7 pedestrian detection network model with other existing pedestrian object detection network models on the INRIA dataset for pedestrian objects with different degrees of overlap.

**Figure 17:**
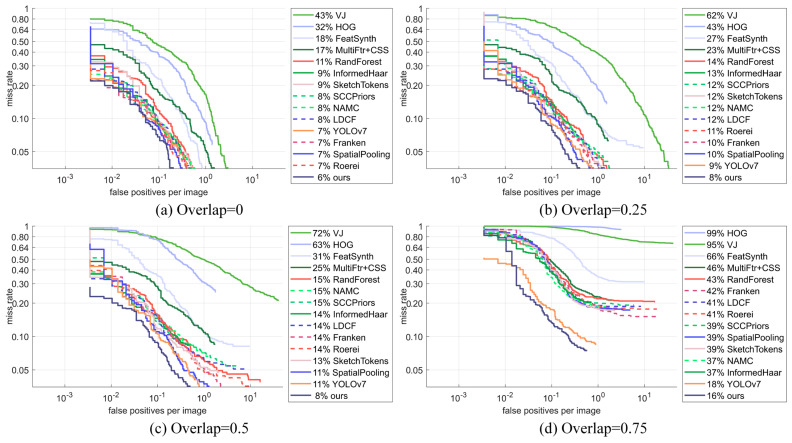
*MR*-*FPPI* curves for subsets with different degrees of overlapping.

As can be seen from the results of the comparative experiments in Table 5, the improved YOLOv7 pedestrian detection network model in this paper achieved excellent performance in all subsets compared with other existing pedestrian object detection networks in terms of the *MR*^−2^ evaluation indexes for the four subsets with overlap degrees of 0, 0.25, 0.5 and 0.75. In the detection of non-overlapping pedestrian object subsets on the INRIA dataset, the *MR*^−2^ of the original YOLOv7 was 7.31%, and the *MR*^−2^ of the improved YOLOv7 model was reduced by 1.3%, reaching 6.01%, which outperforms the existing pedestrian detection algorithms of Franken, SpatialPooling and Roerei compared to the pre-improvement period. In the subsets with overlapping degrees of 0.25 and 0.5, the *MR*^−2^ were 7.74% and 8.33%, respectively, which were reduced by 1.16% and 2.6% compared to the *MR*^−2^ pre-improvement. The *MR*^−2^ of the improved YOLOv7 model was 15.94% in the subset of 0.75 degree of overlap; compared with the pre-improvement *MR*^−2^, this was 2.25% lower. Compared with the other pedestrian detection network models compared in this paper, it was 21.1% lower than the best-performing InformedHaar model in the subset with an overlap degree of 0.75. In order to observe the comparison results more intuitively, the *MR*-*FPPI* curves under different subsets of overlap degree are plotted, as shown in Figure 17. Through the performance comparison mentioned above, the improved YOLOv7 model has excellent performance for pedestrian objects with different overlapping situations, and the recognition performance for severely overlapped objects with an overlap degree of 0.75 is particularly significant.

In order to better understand the region of interest of neural networks in recognizing pedestrian images and to improve the interpretability of the models, this paper uses the Gradient-weighted Class Activation Mapping (Grad-CAM) [39] to generate class activation maps for gradient information visualization of the baseline and improved models, which is achieved by sending the output results back to the network structure as a loss and recording the forward-propagating feature layer and the back-propagating gradient maps of the corresponding layer structure. Then, the gradient map is globally averaged pooling, and the global average pooling result is used as a weight multiplied by the corresponding feature layer. The larger the weight is, the more the network structure pays more attention to the prediction of the feature layer. In this paper, the Grad-CAM map is drawn by the last feature layer used for prediction, and the overall attention of the image is obtained, so as to generate the heat map for the important image regions. The heat intensity distribution reflects the distribution of the region of interest in the model, to help better understand the network.

**Figure 18:**
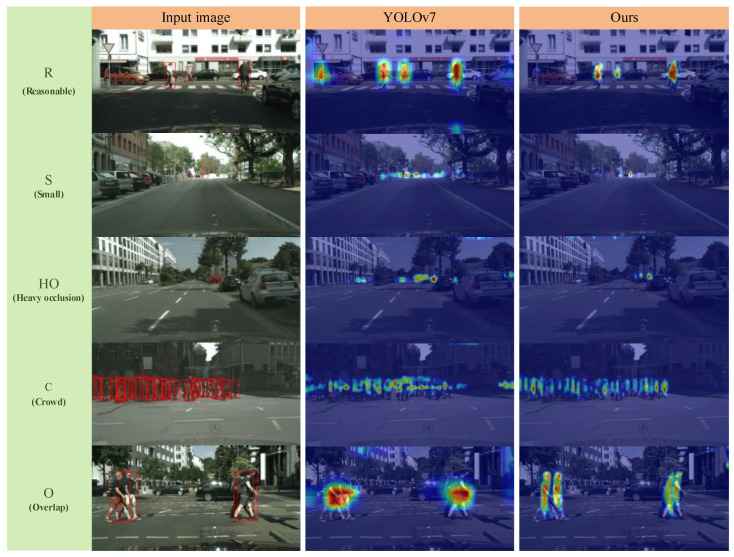
Grad-CAM graphs under different subsets.

For the purpose of comparing the improvement effect of the model more intuitively, the gradient information visualization results of the YOLOv7 model and the improved YOLOv7 model on different subsets of images of the Citypersons dataset were compared by using the Grad-CAM method, as shown in Figure 18.

Based on the visualization results of the heat map distribution in the Grad-CAM graph, it can be seen that for the images of the Reasonable subset in the graph, the improved YOLOv7 model in this paper focuses more on pedestrian objects. Compared with the original YOLOv7 model, the improved model reduces the attention to redundant information in the image. In addition, YOLOv7 in the graph produces misjudgments of pedestrians at the overlap of road signs and vehicles on the left side of the image. As the improved model in this paper can learn more concentrated feature information and efficiently exclude invalid contextual information, it effectively avoids the occurrence of misjudgments.

For the image of the small subset in the figure, the improved model accurately focuses on small pedestrian objects. Although the YOLOv7 model before improvement also focuses on small object pedestrians in the image, compared to the improved model, the pre-improved YOLOv7 model does not pay as much attention to pedestrians as the improved model set, and YOLOv7 also shows excessive attention to the surrounding area without pedestrian objects. For the image of the Heavy occlusion subset in the figure, the improved model pays good attention to the pedestrian positions severely obstructed by vehicles, and also reduces excessive attention to the surrounding areas without pedestrians. For the image of crowded pedestrians in the figure, the improved model shows a more pronounced color transition in the heat map of the crowded pedestrian object, whereas YOLOv7 shows a very chaotic performance.

For the images of the Overlap subset in the figure, the improved model also pays good attention to the overlapping pedestrian objects blocked by pedestrians, and the heat map between pedestrians has a very obvious color transition, indicating that the improved model can distinguish overlapping pedestrian objects well. In summary, through the analysis of the heat map, the improved YOLOv7 model will greatly improve its detection ability for reasonable objects, small objects, severely occluded objects, crowded objects and overlapping objects of pedestrians, which can effectively reduce the false detection rate and missed detection rate of the model in pedestrian object detection tasks.

### 4.5. Validation of Model Generalization

In order to verify the robustness and generalization of the improved YOLOv7 model for pedestrian object detection performance, this paper evaluates the generalization of the trained YOLOv7 model and the improved model without any fine-tuning directly on the Reasonable subset of the ETH dataset by end-to-end training under the Citypersons dataset and the INRIA dataset, respectively.

According to Table 6, in the evaluation results of training under Citypersons dataset and testing under the ETH dataset, the improved YOLOv7 model decreased by 0.73% compared with the *MR*^−2^ before improvement. In the evaluation results of training under the INRIA dataset and testing under the ETH dataset, the improved YOLOv7 model decreased by 12.69% compared with the *MR*^−2^ before improvement. This indicates that our model has learned more general feature representations during the training process on different datasets, so that it can achieve better performance than the original YOLOv7 model on the ETH dataset. At the same time, it can be observed that there is a significant difference in performance improvement when testing the ETH dataset based on different training sets. This is because the overall weather environment and lighting of the Citypersons dataset are darker than those of the INRIA dataset and the ETH dataset, and it contains more small target pedestrians as well as occluded and crowded pedestrians. Therefore, the reason for the significant difference in model performance improvement may be that the style difference between the INRIA dataset and the ETH dataset is smaller than that of the Citypersons dataset, thus having a certain impact on the performance of the model. This also provides ideas for our future research directions. In the future, we need to further study how to better handle the differences between datasets and thus better improve the generalization ability of the model.

## 5. Conclusions

In order to reduce missed and false detections in pedestrian object detection, this paper proposes an improved method based on the YOLOv7 object detection network, which improves the model’s perception ability of pedestrian objects in the input image and modeling ability of pedestrians with different postures, by using the CBAM and DCNv2 in the two feature extraction ELAN modules of the backbone feature extraction network. It also effectively eliminates redundant contextual information around pedestrian objects, allowing the model to learn more concentrated feature representations. In addition, this paper replaces the detection head of YOLOv7 with a DyHead detection head with attention mechanism, which combines scale perception, spatial perception and task perception, and greatly improves the performance of the model in multi-scale scenarios. The improved models in the Citypersons dataset and INRIA dataset achieved good performance improvements compared with the previous model, which effectively proves that the improved method in this paper has good generalization performance in pedestrian detection tasks. In the Citypersons dataset, the improved YOLOv7 model proposed in this paper has the lowest *MR*^−2^ for all subsets of pedestrian objects compared to the original YOLOv7 model, as well as the Faster RCNN, SSD, YOLOv5 and YOLOv8 models. In comparison with other existing pedestrian object detection models in the INRIA dataset, the improved YOLOv7 model also outperforms most of them in terms of performance. By using different subset division methods on the Citypersons dataset containing more small targets, occluded targets and crowded target pedestrians and the INRIA dataset containing more diverse overlapping targets, it has been targeted and fully proved that the improved model has produced very good results under different conditions. This is due to the model’s effective extraction of features and ability to handle complex scenes. In addition, by training on the Citypersons dataset and the INRIA dataset, the generalization verification is completed on the ETH dataset. The validation results show that the improved model has achieved good performance when tested on other datasets. Overall, all experimental results show that the improved YOLOv7 model proposed in this paper achieved good detection performance for pedestrian object detection. At the same time, the computational complexity and number of parameters of the improved model are also reduced. This research provides a detection method that is more computationally efficient and has fewer parameters, higher accuracy and better generalization for the field of pedestrian target detection. Although the inference speed is slightly reduced, in future work, we will further explore how to better combine different attention mechanisms to improve model performance and inference speed, and better handle the differences between datasets to further improve the generalization ability of the model.

## Figures and Tables

**Table 1:** The dataset constructed in this paper.

Dataset		Citypersons	INRIA	ETH
Train	All	2975	1832	0
	Positive sample	2500	614	0
	Negative sample	475	1218	0
Val (Test)	All	500	288	1804
	Positive sample	441	288	1799
	Negative sample	59	0	5

**Table 2:** Subset division by different occlusion, overlap and height.

Setting	Height	Visibility	Overlap
All	[20, +∞)	[0.2, +∞)	0.5
Reasonable (Overlap = 0.5)	[50, +∞)	[0.65, +∞)	0.5
Small	[50, 75]	[0.65, +∞)	0.5
Heavy occlusion	[50, +∞)	[0.2, 0.65]	0.5
Overlap = 0	[50, +∞)	[0.65, +∞)	0
Overlap = 0.25	[50, +∞)	[0.65, +∞)	0.25
Overlap = 0.75	[50, +∞)	[0.65, +∞)	0.75

**Table 3:** Performance comparison of different improvement schemes for the CBAM, DCNv2 and DyHead modules.

Model			AP_50_	AP_50:95_	MR^−2^				FPS
+CBAM	+DCNv2	+DyHead			R	S	HO	A	
YOLOv7(Baseline)			53.8%	36.8%	22.58%	39.50%	53.91%	47.12%	161
√			54.2%	36.8%	21.03%	36.82%	52.71%	46.06%	143
√	√		55.6%	37.6%	20.69%	37.33%	52.41%	45.66%	115
√	√	√	**57.4%**	**39.1%**	**19.02%**	**32.69%**	**50.53%**	**44.09%**	84

In the table, R, S, HO and A respectively represent the Reasonable, Small, Heavy Occlusion and All subsets.

**Table 4:** Performance comparison with other generalized object detection network models.

Model	MR^−2^				Parameters	GFLOPS
	Reasonable	Small	Heavy Occlusion	All		
Faster-RCNN	31.97%	50.53%	67.31%	56.43%	41.3 M	183.3
SSD	33.19%	56.94%	55.12%	56.98%	24.4 M	87.5
YOLOv5l-7.0	27.20%	48.18%	60.02%	52.05%	46.1 M	107.6
YOLOv7	22.58%	39.50%	53.91%	47.12%	36.5 M	103.2
YOLOv8m	27.41%	50.30%	58.82%	52.22%	25.8 M	78.7
YOLOv8l	26.10%	48.56%	60.03%	51.69%	43.6 M	164.8
Ours	**19.02%**	**32.69%**	**50.53%**	**44.09%**	36.7 M	88.5

**Table 5:** Performance comparison with other existing pedestrian object detection network models.

Model	MR^−2^			
	Overlap = 0	Overlap = 0.25	Overlap = 0.5	Overlap = 0.75
VJ [27]	42.68%	62.46%	72.48%	95.01%
HOG [1]	31.50%	43.04%	63.49%	99.32%
FeatSynth [28]	18.23%	27.01%	30.88%	66.38%
MultiFtr + CSS [29]	17.15%	22.59%	24.74%	45.61%
RandForest [30]	10.54%	13.66%	15.37%	42.64%
InformedHaar [31]	9.27%	12.90%	14.43%	37.04%
SketchTokens [32]	8.62%	11.69%	13.32%	38.92%
SCCPriors [33]	8.38%	12.32%	14.54%	39.28%
NAMC [34]	8.17%	11.64%	15.11%	37.48%
LDCF [35]	8.12%	11.61%	13.79%	41.06%
**YOLOv7** [14]	7.31%	9.35%	10.93%	18.19%
Franken [36]	6.88%	10.39%	13.70%	41.83%
SptialPooling [37]	6.63%	9.96%	11.22%	39.18%
Roerei [38]	6.56%	10.78%	13.53%	40.68%
**Ours**	**6.01%**	**7.74%**	**8.33%**	**15.94%**

**Table 6:** Validation of model generalization.

Train		Test	Model	MR^−2^
Citypersons	→	ETH	YOLOv7	52.71%
			Ours	51.98%
INRIA	→	ETH	YOLOv7	59.67%
			Ours	46.98%

## Data Availability

The data underlying the results presented in this paper are not publicly available at this time but may be obtained from the authors upon reasonable request.

## References

[1] Dalal N., Triggs B. Histograms of Oriented Gradients for Human Detection. Proceedings of the 2005 IEEE Computer Society Conference on Computer Vision and Pattern Recognition (CVPR’05).

[2] Lowe D.G. Object recognition from local scale-invariant features. Proceedings of the Seventh IEEE International Conference on Computer Vision.

[3] Cortes C., Vapnik V. (1995). Support-vector networks. Mach. Learn..

[4] Freund Y., Schapire R.E. (1997). A Decision-Theoretic Generalization of On-Line Learning and an Application to Boosting. J. Comput. Syst. Sci..

[5] Girshick R., Donahue J., Darrell T., Malik J. Rich feature hierarchies for accurate object detection and semantic segmenta-tion. Proceedings of the IEEE Conference on Computer Vision and Pattern Recognition.

[6] Girshick R. Fast R-CNN. Proceedings of the 2015 IEEE International Conference on Computer Vision (ICCV).

[7] Ren S., He K., Girshick R., Sun J. (2017). Faster R-CNN: Towards Real-Time Object Detection with Region Proposal Networks. IEEE Trans. Pattern Anal. Mach. Intell..

[8] Liu W., Anguelov D., Erhan D., Szegedy C., Reed S., Fu C.-Y., Berg A.C. (2016). SSD: Single Shot MultiBox Detector. Computer Vision—ECCV 2016, Proceedings of the 14th European Conference, Amsterdam, The Netherlands, 11–14 October 2016.

[9] Redmon J., Divvala S., Girshick R., Farhadi A. You Only Look Once: Unified, Real-Time Object Detection. Proceedings of the 2016 IEEE Conference on Computer Vision and Pattern Recognition (CVPR).

[10] Tang F., Yang F., Tian X. (2023). Long-Distance Person Detection Based on YOLOv7. Electronics.

[11] Wang H., Jin L., He Y., Huo Z., Wang G., Sun X. (2023). Detector–Tracker Integration Framework for Autonomous Vehicles Pedestrian Tracking. Remote Sens..

[12] Zhou C., Yuan J. (2018). Bi-box Regression for Pedestrian Detection and Occlusion Estimation. Computer Vision—ECCV 2018, Proceedings of the 15th European Conference, Munich, Germany, 8–14 September 2018.

[13] Wang X., Xiao T., Jiang Y., Shao S., Sun J., Shen C. Repulsion Loss: Detecting Pedestrians in a Crowd. Proceedings of the 2018 IEEE/CVF Conference on Computer Vision and Pattern Recognition.

[14] Wang C.-Y., Bochkovskiy A., Liao H.-Y.M. YOLOv7: Trainable Bag-of-Freebies Sets New State-of-the-Art for Real-Time Object Detectors. Proceedings of the 2023 IEEE/CVF Conference on Computer Vision and Pattern Recognition (CVPR).

[15] Woo S., Park J., Lee J.-Y., Kweon I.S. (2018). CBAM: Convolutional Block Attention Module. Computer Vision—ECCV 2018, Proceedings of the 15th European Conference, Munich, Germany, 8–14 September 2018.

[16] Zhu X., Hu H., Lin S., Dai J. Deformable convnets v2: More deformable, better results. Proceedings of the IEEE/CVF Conference on Computer Vision and Pattern Recognition.

[17] Dai X., Chen Y., Xiao B., Chen D., Liu M., Yuan L., Zhang L. Dynamic Head: Unifying Object Detection Heads with Attentions. Proceedings of the 2021 IEEE/CVF Conference on Computer Vision and Pattern Recognition (CVPR).

[18] Zhang S., Benenson R., Schiele B. CityPersons: A Diverse Dataset for Pedestrian Detection. Proceedings of the 2017 IEEE Conference on Computer Vision and Pattern Recognition (CVPR).

[19] Ess A., Leibe B., Van Gool L. Depth and Appearance for Mobile Scene Analysis. Proceedings of the 2007 IEEE 11th International Conference on Computer Vision.

[20] Jocher G., Chaurasia A., Qiu J. Ultralytics YOLO, Version 8.0.0; Ultralytics, USA; 2023. https://github.com/ultralytics/ultralytics.

[21] He K., Zhang X., Ren S., Sun J. (2015). Spatial Pyramid Pooling in Deep Convolutional Networks for Visual Recognition. IEEE Trans. Pattern Anal. Mach. Intell..

[22] Wang C.-Y., Mark Liao H.-Y., Wu Y.-H., Chen P.-Y., Hsieh J.-W., Yeh I.-H. CSPNet: A New Backbone that can Enhance Learning Capability of CNN. Proceedings of the 2020 IEEE/CVF Conference on Computer Vision and Pattern Recognition Workshops (CVPRW).

[23] Lee Y., Hwang J., Lee S., Bae Y., Park J. An Energy and GPU-Computation Efficient Backbone Network for Real-Time Object Detection. Proceedings of the 2019 IEEE/CVF Conference on Computer Vision and Pattern Recognition Workshops (CVPRW).

[24] Dai J., Qi H., Xiong Y., Li Y., Zhang G., Hu H., Wei Y. Deformable convolutional networks. Proceedings of the IEEE International Conference on Computer Vision.

[25] Dollar P., Wojek C., Schiele B., Perona P. (2012). Pedestrian Detection: An Evaluation of the State of the Art. IEEE Trans. Pattern Anal. Mach. Intell..

[26] Jocher G. (2020). YOLOv5 by Ultralytics.

[27] Viola P., Jones M.J. (2004). Robust real-time face detection. Int. J. Comput. Vis..

[28] Bar-Hillel A., Levi D., Krupka E., Goldberg C. (2010). Part-based feature synthesis for human detection. Computer Vision–ECCV 2010, Proceedings of the 11th European Conference on Computer Vision, Heraklion, Crete, Greece, 5–11 September 2010.

[29] Walk S., Majer N., Schindler K., Schiele B. New features and insights for pedestrian detection. Proceedings of the 2010 IEEE Computer Society Conference on Computer Vision and Pattern Recognition.

[30] Marin J., Vazquez D., Lopez A.M., Amores J., Leibe B. Random Forests of Local Experts for Pedestrian Detection. Proceedings of the 2013 IEEE International Conference on Computer Vision.

[31] Zhang S., Bauckhage C., Cremers A.B. Informed Haar-Like Features Improve Pedestrian Detection. Proceedings of the 2014 IEEE Conference on Computer Vision and Pattern Recognition.

[32] Lim J.J., Zitnick C.L., Dollar P. Sketch Tokens: A Learned Mid-level Representation for Contour and Object Detection. Proceedings of the 2013 IEEE Conference on Computer Vision and Pattern Recognition.

[33] Yang Y., Wang Z., Wu F. Exploring Prior Knowledge for Pedestrian Detection. Proceedings of the British Machine Vision Conference 2015.

[34] Ţoca C., Ciuc M., Pătraşcu C. Normalized Autobinomial Markov Channels For Pedestrian Detection. Proceedings of the British Machine Vision Conference.

[35] Nam W., Dollar P., Han J.H. (2014). Local decorrelation for improved pedestrian detection. Adv. Neural Inf. Process. Syst..

[36] Mathias M., Benenson R., Timofte R., Gool L.V. Handling Occlusions with Franken-Classifiers. Proceedings of the 2013 IEEE International Conference on Computer Vision.

[37] Paisitkriangkrai S., Shen C., Hengel A. (2014). Strengthening the effectiveness of pedestrian detection with spatially pooled features. Computer Vision–ECCV 2014, Proceedings of the 13th European Conference, Zurich, Switzerland, 6–12 September 2014.

[38] Benenson R., Mathias M., Tuytelaars T., Van Gool L. Seeking the Strongest Rigid Detector. Proceedings of the 2013 IEEE Conference on Computer Vision and Pattern Recognition.

[39] Selvaraju R.R., Cogswell M., Das A., Vedantam R., Parikh D., Batra D. Grad-cam: Visual explanations from deep networks via gradient-based localization. Proceedings of the IEEE International Conference on Computer Vision.

